# Shift work, clinically significant sleep disorders and mental health in a representative, cross-sectional sample of young working adults

**DOI:** 10.1038/s41598-022-20308-2

**Published:** 2022-09-28

**Authors:** Amy C. Reynolds, Bastien Lechat, Yohannes Adama Melaku, Kelly Sansom, Brandon W. J. Brown, Meagan E. Crowther, Sian Wanstall, Kathleen J. Maddison, Jennifer H. Walsh, Leon Straker, Robert J. T. Adams, Nigel McArdle, Peter R. Eastwood

**Affiliations:** 1 Flinders Health and Medical Research Institute (Sleep Health), Flinders University, Mark Oliphant Building, 5 Laffer Drive, Bedford Park, Adelaide, South Australia 5039 Australia; 2 Department of Pulmonary Physiology and Sleep Medicine, West Australian Sleep Disorders Research Institute, Sir Charles Gairdner Hospital, Perth, WA Australia; 3 School of Human Sciences, Centre for Sleep Science, The University of Western Australia, Perth, WA Australia; 4 Appleton Institute, CQ University Australia, Adelaide, South Australia Australia; 5 School of Allied Health, Curtin University, Perth, WA Australia; 6 Flinders Health and Medical Research Institute, Flinders University, Bedford Park, Adelaide, Australia

**Keywords:** Human behaviour, Epidemiology

## Abstract

Mental health conditions confer considerable global disease burden in young adults, who are also the highest demographic to work shifts, and of whom 20% meet criteria for a sleep disorder. We aimed to establish the relationship between the combined effect of shift work and sleep disorders, and mental health. The Raine Study is the only longitudinal, population-based birth cohort in the world with gold-standard, Level 1 measurement of sleep (polysomnography, PSG) collected in early adulthood. Participants (aged 22y) underwent in-laboratory PSG and completed detailed sleep questionnaires. Multivariable adjusted robust linear regression models were conducted to explore associations with anxiety (GAD7) and depression (PHQ9), adjusted for sex, health comorbidities, and work hours/week. Data were from 660 employed young adults (27.3% shift workers). At least one clinically significant sleep disorder was present in 18% of shift workers (day, evening and night shifts) and 21% of non-shift workers (*p* = 0.51); 80% were undiagnosed. Scores for anxiety and depression were not different between shift and non-shift workers (*p* = 0.29 and *p* = 0.82); but were higher in those with a sleep disorder than those without (*Md(IQR)* anxiety: 7.0(4.0–10.0) vs 4.0(1.0–6.0)), and depression: (9.0(5.0–13.0) vs 4.0(2.0–6.0)). Considering evening and night shift workers only (i.e. excluding day shift workers) revealed an interaction between shift work and sleep disorder status for anxiety (*p* = 0.021), but not depression (*p* = 0.96), with anxiety scores being highest in those shift workers with a sleep disorder (*Md(IQR)* 8.5(4.0–12.2). We have shown that clinical sleep disorders are common in young workers and are largely undiagnosed. Measures of mental health do not appear be different between shift and non-shift workers. These findings indicate that the identification and treatment of clinical sleep disorders should be prioritised for young workers as these sleep disorders, rather than shift work per se*,* are associated with poorer mental health. These negative mental health effects appear to be greatest in those who work evening and/or night shift and have a sleep disorder.

## Introduction

Mental health conditions are a leading contributor to global disease burden in young adults^1^, of whom one in five are shift workers, and up to 20% have at least one clinically significant sleep disorder^2^. Shift work has previously been associated with poor mental health, including anxiety and depression^3^. However, despite high rates of participation in shift work^4^, and high prevalence and burden of mental health conditions^1^, young adults are globally underrepresented in studies of the relationship between shift work and mental health.

Shift work refers to schedules which fall outside more traditional hours (0900–1800), and typically induces a degree of circadian disruption due to a requirement to work when the body is biologically primed to sleep^5^. This circadian disruption is thought to play an important role in the relationship between shift work and poor mental health^6–8^. However, what is commonly overlooked is that clinical sleep disorders in young adult shift workers could influence this relationship. Day, evening and night shift schedules are all associated with sleep disorders^9–11^ in existing literature, predominantly in middle-aged and older workers. Further, all common clinical sleep disorders, including insomnia, obstructive sleep apnea (OSA) and restless legs syndrome (RLS), are associated with poor mental health. For example, insomnia precedes development of mental illness^12^ and can increase the risk of relapse of mental health disorders, including depression^13^. Untreated OSA is also associated with incident major depressive disorder^14^, and women with RLS are more likely to develop incident clinical depression^15^.

Despite relationships between poorer mental health and both shift work and sleep disorders, little is understood about the interaction between shift work and common clinical sleep disorders in young adults. Importantly, if an association were present, it may be possible to design early diagnosis and intervention strategies for sleep disorders that could benefit the mental health of young shift workers.

This study aimed to investigate, for the first time in a community sample of young adults, the association between shift work, clinical sleep disorders and mental health.

## Methods

### Study design and population

We included participants recruited as part of the Raine Study, one of the largest, multigenerational prospective cohorts of pregnancy, childhood, adolescence and adulthood to be conducted in the world. The participants in this longitudinal prospective cohort (known as Gen 2) have been followed since prenatal screening in 1989–1991 at the largest public obstetric hospital in Western Australia^16^. Measurements commenced in-utero, and participants have been studied regularly since recruitment. All research was performed in accordance with relevant guidelines, informed consent was obtained from all participants, and research was conducted in accordance with the requirements of the Declaration of Helsinki.

### Cohort attrition, follow-up information and representativeness

**Figure 1:**
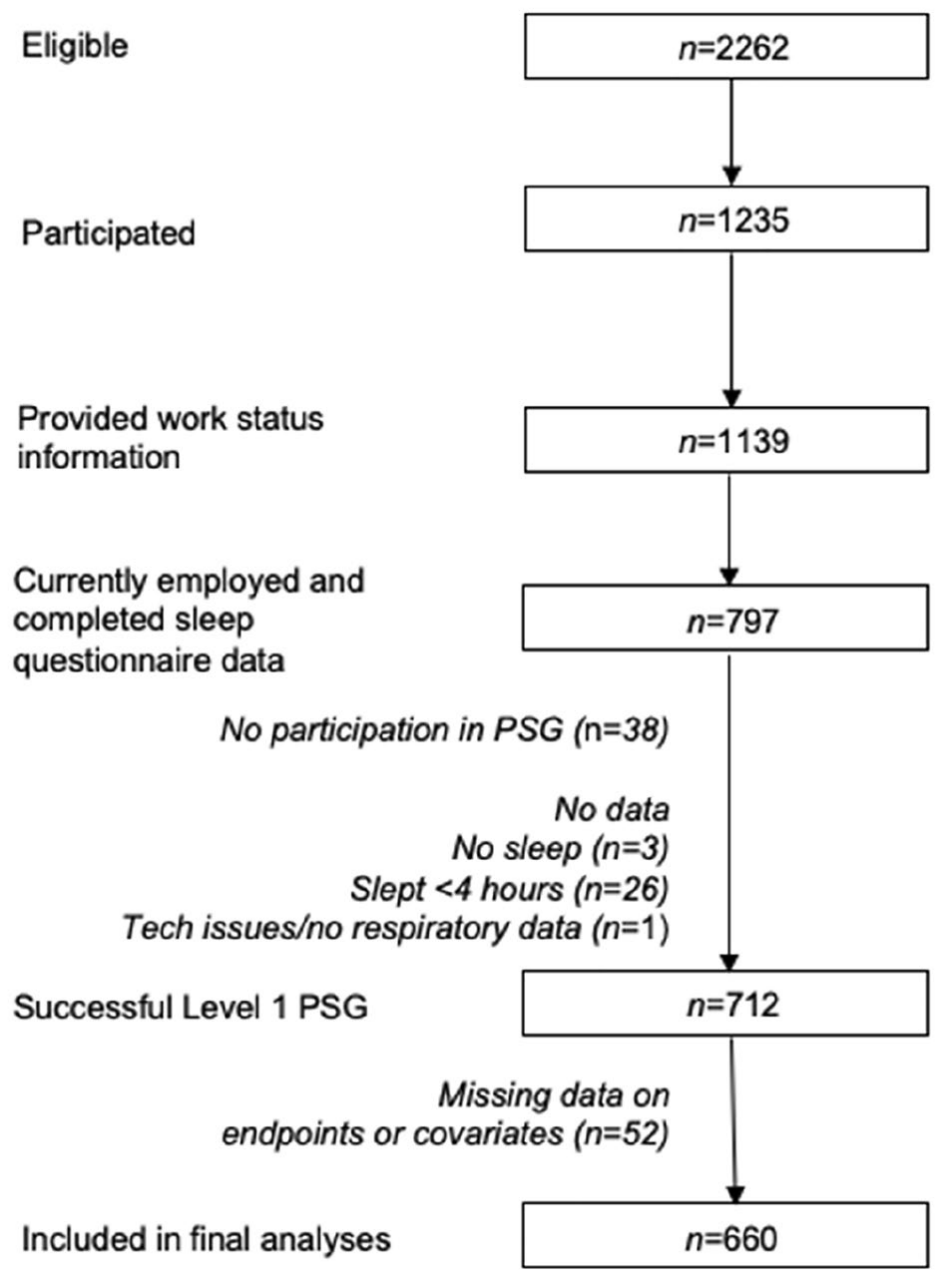
Study flow diagram. PSG = polysomnography.

Between March 2012 to June 2014 (the year of their 22^nd^ birthday), participants were invited to participate in a comprehensive follow-up (referred to as Gen2-22)^17^ which received ethical approval (University of Western Australia, RA/4/1/5202). Figure 1 shows the profile of participants at the time of participation in the Raine Study Gen2-22 year follow up. The overall prevalence rates of these sleep disorders in the young adult population, including participants who were not currently employed, are described elsewhere^2^. Of the total participants who participated in the follow-up (*n* = 1,235), 1,139 (92.2%) provided work status information; of these, 932 (81.8%) were currently employed, and 797 (70.0%) also completed the follow-up sleep questionnaire. Of those participants who completed the sleep questionnaire 712 (89.3%) completed a successful PSG; 52 participants (7.3%) had incomplete data on endpoints or relevant covariates, and were not included in the analyses, leaving *n* = 660 participants for inclusion in analyses. Characteristics of the sample can be found in Table 1. Additional characteristics of the sample according to shift work status can be found in Supplementary Table 1.

**Table 1:** Demographic characteristics of participants overall, and by shift work status.

	Overall (*n* = 660)	Non-shift work (*n* = 480)	Shift work (*n* = 180)	*p*
Age (range)	21.9 (21.7–22.3)	22.0 (21.7–22.3)	21.9 (21.6–22.2)	0.32
Sex (female)	349 (53)	260 (54)	89 (49)	0.28
BMI	24.0 (21.9–27.1)	24.1 (21.9–26.9)	23.9 (21.8 – 28.5)	0.74
Sleep disorder^a,b^	132 (20)	99 (21)	33 (18)	0.51
Chronic insomnia	98 (15)	72 (15)	26 (15)	0.85
Clinically significant OSA	32 (4.8)	26 (5.4)	6 (3.3)	0.27
Restless legs syndrome	6 (0.9)	5 (1.1)	1 (0.6)	1.00
Education				0.15
Secondary school	304 (48)	214 (47)	90 (51)	
TAFE or college	137 (22)	101 (22)	36 (20)	
University	179 (28)	135 (29)	44 (25)	
Other	16 (2.5)	8 (1.7)	8 (4.5)	
(missing, n)	24	22	2	
Presence of comorbidities	250 (38)	184 (38)	66 (37)	0.69
Marital status				0.80
Not married	502 (79)	362 (80)	140 (79)	
Registered marriage or defacto	131 (21)	93 (20)	38 (21)	
(missing, n)	27	25	2	
Income category^c^				0.49
Low	322 (51)	228 (51)	94 (54)	
Medium	237 (38)	177 (39)	60 (34)	
High	67 (11)	46 (10)	21 (12)	
(missing, n)	34	29	5	
Paid work hours/week	35 (16–40)	36 (16–40)	30 (17–40)	0.90
Years in current job	1.58 (0.6–3.3)	1.7 (0.6–3.4)	1.3 (0.5–3.0)	0.36

Data are median (IQR) or n (%) unless otherwise specified. Differences between shift work groups were determined using Pearson’s chi-squared test, Wilcoxon rank sum test, or Fisher’s exact test.

^a^Sleep disorder columns will not add to totals, as participants could meet criteria for more than one common clinically significant sleep disorder.

^b^Sleep disorders are expressed as n(%).

^c^Income categories in $AUD after tax include low (< $32,000 per annum), medium ($32,000—$64,999 per annum), and high (≥ $65,000).

The representativeness of the cohort has been detailed elsewhere^16^. Briefly, representativeness was consistently examined from eligibility and consent, at birth, and at the 8, 14, 17, 20 and 22 year follow ups. The cohort is consistently similar to Western Australian families (and comparable young adults at the 20- and 22-year follow ups). The only differences at the 17-year follow up were a higher proportion of participants employed in clerical/retail, more with > 40 working hours per week, and more participants with higher incomes. Other indicators of socioeconomic status were comparable, and there was no evidence of attrition bias across the majority of infant characteristics, although a decline in infants identified as Aboriginal and Torres Strait Islander^16^. Additionally, comparison against Western Australian Census data confirmed that participants who completed sleep studies in the Gen2-22 follow-up were broadly representative of 22-year olds in West Australia^2^. An additional comparison of select occupational and education data in the subset of the young working adults who completed sleep studies and were currently employed showed comparable distribution across occupation categories against Western Australian Census data, and a smaller proportion of participants in the ‘low’ income bracket, in line with the 17-year follow-up (see Supplementary Table 2).

#### Shift work status

Participants were asked about their current employment. Shift work status was classified from a yes/no response to the question ‘Are you a shift worker?’*.* If participants responded that they were a shift worker, they were also asked to indicate the shift schedule(s) they worked most commonly; day, evening, or night shift. Participants could select multiple options as appropriate to their working arrangements. All participants who identified as shift workers were included in the primary analysis.

#### Sleep disorder status

Participants provided informed consent and underwent an overnight laboratory-based sleep study (polysomnography, [PSG]) at the Centre for Sleep Science, The University of Western Australia^2^. Sleep studies were conducted by trained sleep technologists using Grael amplifier technology (Compumedics, Abbostford, Victoria, Australia) and recordings were analysed according to guidelines established by the American Academy of Sleep Medicine (2012)^18^ to detect presence and severity of obstructive sleep apnea (OSA) as defined by the apnea hypopnea index (AHI).

Chronic insomnia was determined using the Pittsburgh Sleep Symptom Questionnaire (PSSQ_I)^19^, with a modified symptom duration of ≥ 3 months to align with current diagnostic criteria for chronic insomnia. Clinically significant Restless Legs Syndrome (RLS) was identified with the diagnostic criteria identified by the International Restless Legs Syndrome Study Group (IRLSSG)^20^, specifically if participants reported all four RLS symptoms and experienced these symptoms ≥ 5 times/month^2^. Sleepiness was measured according to the Epworth Sleepiness Scale (ESS)^21^.

Overall, participants were determined to have at least one clinically significant sleep disorder if they met criteria for ≥ 1 of the following: *clinically significant OSA* (either an AHI ≥ 15 events/hour or OSA syndrome: AHI ≥ 5 events/hour and ESS ≥ 11), *chronic insomnia*, or *RLS*. Participants could meet criteria for more than one clinically significant sleep disorder.

#### Choice of primary measure

Continuous scores on the Patient Health Questionnaire (PHQ)-9 and the Generalised Anxiety Disorder (GAD)-7 scales were used to assess depression and anxiety symptomatology respectively. The PHQ-9 comprises nine items, and is intended to screen for depression^22^. Scores range from 0–27, with major depression indicated by a score of ≥ 10. The GAD-7 consists of seven items used to screen for anxiety^23^. Scores range from 0–21, with a score of ≥ 10 indicating generalised anxiety disorder. The PHQ-9 is widely used in primary care settings, other medical contexts and in research, with good diagnostic accuracy compared to semi structured diagnostic interviews^24^. The GAD-7 similarly shows good sensitivity and specificity for identifying generalised anxiety disorder (score ≥ 10) when compared with structured psychiatric interviews^23^.

The GAD-7 and PHQ-9 were selected based on their availability for use, the wide utilisation in population health and clinical studies, the availability of normative data for comparison, and strong sensitivity and specificity for identifying clinically significant anxiety and depression.

#### Assessment of covariates

Sex is reflected as biological status at birth, with only binary (male or female) responses. Participants were coded (yes/no) as having current comorbidities if they indicated that they were currently experiencing effects from any of the following conditions which have known associations with the endpoints of interest; *arthritis/joint problems, back pain, neck pain, migraine or severe headache, menstrual problems, heart condition, eating disorder/weight problems, diabetes, coeliac disease, chronic respiratory problems, or asthma*. Self-reported typical work hours expected in a week were included as a covariate due to known associations between work hours and mental health endpoints.

#### Statistical analysis

Data were analysed using RStudio (RStudio Team 2018, Boston MA) and R (R Core Team (2021), Vienna Austria). Participants were included in analyses for mental health endpoints if they completed a successful PSG with ≥ 4 h of sleep, in addition to sleep questionnaires, in order to establish prevalence of at least one common, clinically significant sleep disorder. Robust regression analyses *(MASS)* with *M* estimators to account for the influence of outliers and non-normality of the continuous mental health endpoints were conducted to examine the main effects and interaction of work schedule (shift *vs* non-shift) and sleep disorder status (*yes* or *no*) on continuous anxiety and depression scores. Models were adjusted for sex, comorbidities, and work hours per week based on a priori established relationships with the endpoints of interest.

Secondary robust regression analyses were undertaken to determine whether an interaction with sleep disorders on mental health endpoints was present when the reported shift work schedule overlapped with evening or night sleep opportunity. In these analyses day shift workers, and shift workers who did not report their shift schedules, were excluded from the ‘shift work’ group and only those who worked evening (1500-0000) and/or night shift (2200-0800) were considered.

## Results

### Prevalence of common clinical sleep disorders by shift work status

At least one clinical sleep disorder was present in 20% of the young workers in this community sample^2^ with similar prevalence estimates between shift and non-shift workers (18% vs 21% respectively, *p* = 0.51). Chronic insomnia was the most common sleep disorder (15%), followed by clinically significant OSA (4.8%) and clinically significant RLS (0.9%). The majority (80%) of participants who met criteria for a clinical sleep disorder had never been told by a health professional that they have a sleep disturbance.

### Mental health by shift work and sleep disorder status

Anxiety (*p* = 0.29), and depression (*p* = 0.83) were not different between shift and non-shift workers (Table 2). In contrast, scores for anxiety and depression (both *p* < 0.001) were significantly greater in workers who had a clinical sleep disorder. Further, a significantly higher proportion of workers with a clinical sleep disorder exceeded the threshold for moderate or severe anxiety and depression (Table 2).

**Table 2:** Mental health outcomes by shift work and sleep disorder status.

	Overall (*n* = 660)	Non-shift worker (*n* = 480)	Shift worker (*n* = 180)	*p*	No sleep disorder (*n* = 528)	Any sleep disorder (*n* = 132)	*p*
GAD-7 total score (0–21)	4.0 (2.0–7.0)	4.0 (1.0–7.0)	4.0 (2.0–7.0)	0.29	4.0 (1.0–6.0)	7.0 (4.0–10.0)	< 0.001
PHQ-9 total score (0–27)	4.0 (2.0–8.0)	4.0 (2.0–8.0)	4.0 (2.0–7.0)	0.83	4.0 (2.0–6.0)	9.0 (5.0–13.0)	< 0.001
GAD-7 cut points*				0.97			< 0.001
Normal	381 (58)	278 (58)	103 (57)		335 (63)	46 (35)	
Mild anxiety	191 (29)	139 (29)	52 (29)		145 (27)	46 (35)	
Moderate anxiety	59 (8.9)	43 (9.0)	16 (8.9)		35 (6.6)	24 (18)	
Severe Anxiety	29 (4.4)	20 (4.2)	9 (5.0)		13 (2.5)	16 (12)	
PHQ-9 cut points*				0.59			< 0.001
Normal	353 (53)	256 (53)	97 (54)		324 (61)	29 (22)	
Minimal symptoms	193 (29)	135 (28)	58 (32)		151 (29)	42 (32)	
Minor depression, or major depression (mild)	73 (11)	57 (12)	16 (8.9)		37 (7.0)	36 (27)	
Major depression (moderate)	35 (5.3)	27 (5.6)	8 (4.4)		15 (2.8)	20 (15)	
Major depression (severe)	6 (0.9)	5 (1.0)	1 (0.6)		1 (0.2)	5 (3.8)	

Data are median (IQR) or n (%) unless otherwise specified. Differences between shift work groups were determined using Pearson’s chi-squared test, Wilcoxon rank sum test, or Fisher’s exact test. GAD-7, generalised anxiety disorder (GAD)-7 anxiety scale; PHQ-9, patient health questionnaire (PHQ)-9 depression; *, cutpoints determined according to existing literature^25^.

**Figure 2:**
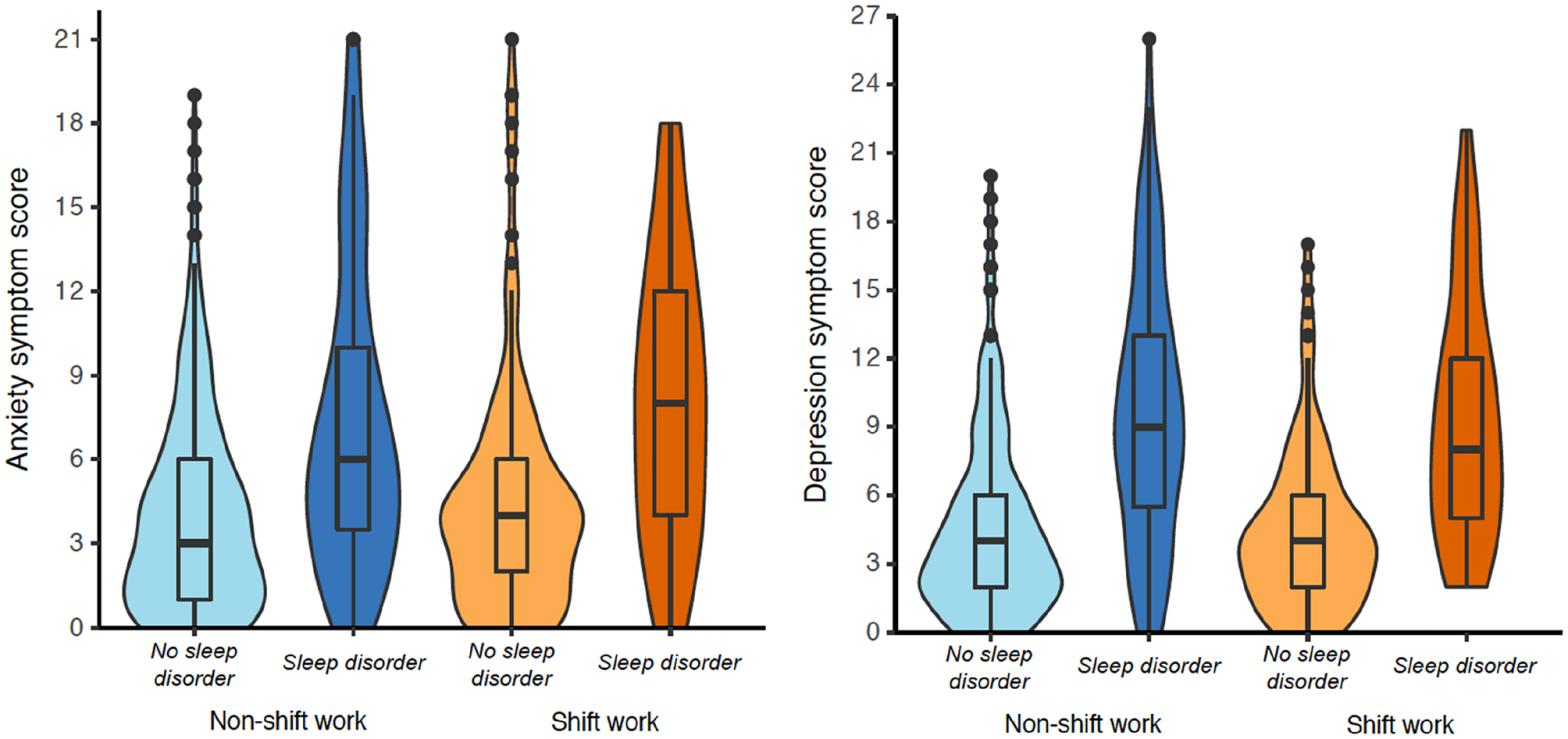
Anxiety symptom score (left) and depression symptom score (right) by shift work and sleep disorder status**.** Violin plots reflect the density (width) and range (height) of scores within each category of work type and sleep disorder status. Inset boxplots depict the median and interquartile ranges. Black dots reflect outlier values. Anxiety is measured with the GAD-7 (range from 0–21), and depression is measured with the PHQ-9 (range from 0–27).

This finding that, regardless of shift work status, the presence of clinical sleep disorders was associated with higher levels of anxiety and depression remained evident when using adjusted models with main and interactive effects (Table 3, Fig. 2). The highest median anxiety scores were observed for shift workers with sleep disorders (Fig. 2). No significant interactions were observed between shift work and sleep disorder status and both anxiety (*p* = 0.064) and depression (*p* = 0.78) scores (Table 3), indicating that anxiety and depression were not significantly worse in workers reporting a combination of both shift work and a clinical sleep disorder.

**Table 3:** Adjusted associations between shift work, sleep disorder status and mental health endpoints.

Predictors	Anxiety			Depression		
	β	95% CI	*p*	β	95% CI	*p*
(Intercept)	2.99	2.29–3.69	** < 0**.**001**	3.71	2.97–4.45	** < 0.001**
Shift work (yes)	0.26	− 0.40–0.93	0.44	− 0.01	-0.71–0.69	0.98
Clinical sleep disorder (yes)	2.42	1.65–3.20	** < 0.001**	4.73	3.91–5.55	** < 0.001**
Shift work*Sleep disorder	1.45	− 0.08– 2.98	0.064	− 0.23	− 1.85–1.38	0.78

Significant values are in [bold].

Models adjusted for sex, health comorbidities, and typical work hours/week. Anxiety is measured with the GAD-7 (range from 0–21), and depression is measured with the PHQ-9 (range from 0–27).

Supplementary Table 3 provides the shift work schedule combinations reported by participants. Considering evening and night shift workers only (i.e. excluding n = 46 day shift workers and *n* = 4 participants who did not report their shift types) revealed an interaction between shift work and sleep disorder status for anxiety (*p* = 0.021), but not depression (*p* = 0.96; see Supplementary Table 4).

## Discussion

We provide new evidence in a community sample of young workers that clinical sleep disorders, rather than shift work, are associated with poorer mental health. The major findings of this study were that (1) 20% of a community-based sample of young workers met criteria for having at least one clinically significant sleep disorder, despite the majority of disorders (80%) being undiagnosed prior to their participation in the study; (2) anxiety and depression scores were higher in those with a sleep disorder, regardless of whether they were a shift worker or not; and (3) the rates of depression (46%) and anxiety (30%) in those with a clinical sleep disorder far exceeded rates in existing, similarly aged populations^26,27^. These findings show that clinical sleep disorders are prevalent in young adult workers, are largely undiagnosed, and are associated with noticeably poorer mental health. This study also indicates that poor mental health in young adult shift workers might reflect the effects of undiagnosed sleep disorders, rather than the effects of shift work.

Our finding of higher anxiety and depression scores in young workers with clinical sleep disorders is consistent with previous findings in other working populations^28,29^. However, prior studies examining the relationship between work, sleep disorders, and mental health commonly include few, or no, young adult workers. Further, existing studies regularly rely on self-reported risk indicators for sleep disorders rather than use of polysomnography to identify OSA, or report findings from specific industries or occupations^28^ rather than prevalence rates in population representative samples. Thus, the current study provides novel insight into these associations in young workers, and importantly, across a representative community sample with both polysomnography and detailed sleep questionnaires. The importance of this approach for a true estimate of sleep disorders is particularly illustrated by the high prevalence of undiagnosed sleep disorders in this sample, which would not have been identified by self-report alone.

Existing literature on the relationship between shift work and mental health is mixed, with strongest prospective associations observed for depression, and for female but not male shift workers^3^. This is commonly attributed to insufficient sleep (or sleep disturbances) and circadian disruption associated with work schedules^6^. Our findings provide a new perspective for young workers, suggesting that undiagnosed sleep disorders are an important consideration in the relationship between work and mental health. Efforts to improve healthy sleep behaviour and environments, optimise shift schedules and utilise alertness or sleep promoting agents are commonly recommended in shift working occupations. We suggest based on these analyses, that there is an additional unmet need for early sleep disorder education, diagnosis and intervention in young shift workers and non-shift workers, particularly as young workers in our sample were largely unaware of their sleep disorder. Secondary analyses suggest this is particularly relevant for shift workers with evening and/or night shift requirements, where a significant interaction between shift work status and sleep disorder status was associated with clinically meaningful anxiety scores on the GAD-7. Consideration should be given to identifying sleep disorders in addition to schedule- or alerting-focussed interventions which could potentially mask undiagnosed sleep disorders, or inappropriately attribute sleepiness and fatigue solely to shift work schedules.

We did not observe an independent association between shift work and either depression or anxiety in the current sample in our primary analysis. This is in agreement with recent studies which suggest that shift work may not be a predictor of poorer mental health status^30,31^, but in contrast to others which suggest negative impacts of shift work on mental health^3^. This is likely in part to be because our sample were relatively early in their careers (*median duration* 1.5 years) and that any associations between shift work and mental health may be dependent on a longer exposure to shift work than observed in our sample. However, it is important to note that increasing time in work is by nature synonymous with increasing age, and thus an increased risk for sleep disorders. Community studies suggest that prevalence of clinically significant sleep disorders increases from 20%^2^ in early adulthood to almost 50% by mid-life^32^. With more time in shift work comes an increased risk of an undiagnosed sleep disorder, which may further influence any associations between shift work and mental health.

The prevalence of clinical sleep disorders which are largely undiagnosed, and the associations with poor mental health, are concerning in this sample of young workers. However, these findings also suggest a promising and novel target for intervention to improve mental health in young adults as treatment for sleep disorders such as OSA and insomnia are well established^33,34^ and are associated with improvements in mental health. Notably, the data on which these findings are based are from studies largely conducted in middle-aged and older adults^33^, and in workers on ‘standard’ (or non-shift) schedules. Similar clinical trials, undertaken in younger adults with sleep disorders, are sorely needed.

A key strength of this study is inclusion of the largest, representative sample of young workers with both shift and non-shift working participants, with Level 1, in-laboratory measurement of sleep disorders along with detailed sleep questions. A limitation of the study is the cross-sectional nature of the data presented, meaning causal relationships between sleep disorders, shift work and mental health could not be determined, although this was not the intent of the analysis. This also means that reverse causality cannot be excluded, and future studies could consider whether pre-existing mental health conditions combined with sleep disorders influence decisions to enter an occupation with shift work requirements. Due to modest numbers of night shift workers with clinical sleep disorders, we were also unable to explore these associations by individual schedule type. However secondary analyses that excluded day shift workers revealed that anxiety scores were significantly worse in those who worked evening and/or night shift and had a sleep disorder. Future collection of shift work, sleep and mental health endpoints in the Raine Study will facilitate further longitudinal analysis of the association between shift work, sleep disorders, and mental health outcomes, and allow for temporal relationships to be explored in this unique, young cohort with robust sleep measurements.

## Conclusion

Mental health conditions reflect a substantial burden of disease in young adulthood^1^. As transition to employment in young adulthood is already recognised as challenging, there is an urgent need for novel, early interventions to support improvements in mental health. Sleep disorders are common in young working individuals, with 20% meeting criteria for a disorder which warrants clinical intervention, and are ultimately treatable if identified and referred appropriately; yet 80% of those living with a clinically significant sleep disorder in our community sample reported no diagnosis of any sleep disturbance.

This study showed that sleep disorders, rather than shift work, were associated with poorer mental health. Further, those who worked evening and/or night shifts and had a sleep disorder had higher anxiety scores, indicative of poorer mental health. We propose that identification and treatment of sleep disorders in this under-recognised group is an important target for early intervention to support mental health in young, working adults. Education, identification and management related to sleep disorders for shift workers should be prioritised both in primary care, and in occupational health settings alongside scheduling and other fatigue risk management priorities in order to manage workplace health and safety risks.

## Supplementary Information

Supplementary Information.

## Data Availability

The data that support the findings of this study are available from the Raine Study but restrictions apply to the availability of these data, which were used under formal scientific review approvals processes for the current study, and so are not publicly available. Data availability is subject to request and with permission of the Raine Study, which has a transparent data access and approvals process found at https://rainestudy.org.au.
