# Supplementary material for: Shift work, clinically significant sleep disorders and mental health in a representative, cross-sectional sample of young working adults
Source: Sci Rep. 2022 Sep 28;12:16255. doi: 10.1038/s41598-022-20308-2 (PMC9519578; doi:10.1038/s41598-022-20308-2)
Supplement: Supplementary file 1 — Supplementary Information. [file 41598_2022_20308_MOESM1_ESM.docx]

**Supplementary Table 1:** Additional characteristics of the sample overall, by shift work status, and by sleep disorder status

|  | **Overall**  (*n*=660) | **Non-shift work**  (*n*=480) | **Shift work**  (*n*=180) | *p* | **No disorder**  (*n*=528) | **Sleep disorder**  (*n*=132) | *p* |
| --- | --- | --- | --- | --- | --- | --- | --- |
| Type of shift^a^ |  |  |  |  |  |  |  |
| *Day (0600 – 1900)* | 125 (19) | · | 125 (69) | · | 108 (20) | 17 (13) | 0·047 |
| *Evening (1500 – 0000)* | 103 (16) | · | 103 (57) | · | 85 (16) | 18 (14) | 0·49 |
| *Night (2200 – 0800)* | 50 (7·6) | · | 50 (28) | · | 41 (7·8) | 9 (6·8) | 0·71 |
| *(no data on shift type)* | 4 (0·01) |  | 4 (2·2) |  |  |  |  |
| Education |  |  |  | 0·15 |  |  | 0·15 |
| *Secondary school* | 304 (48) | 214 (47) | 90 (51) |  | 236 (46) | 68 (54) |  |
| *TAFE/College* | 137 (22) | 101 (22) | 36 (20) |  | 110 (22) | 27 (21) |  |
| *University* | 179 (28) | 135 (29) | 44 (25) |  | 152 (30) | 27 (21) |  |
| *Other* | 16 (2·5) | 8 (1·7) | 8 (4·5) |  | 11 (2·2) | 5 (3·9) |  |
| *(missing, n)* | 24 | 22 | 2 |  | 19 | 5 |  |
| Occupation |  |  |  | <0·001 |  |  | 0·34 |
| *Professional/Managerial* | 108 (16) | 86 (18) | 22 (12) |  | 92 (17) | 16 (12) |  |
| *Clerical* | 112 (17) | 100 (21) | 12 (6·7) |  | 85 (16) | 27 (21) |  |
| *Technical/Trade/Labour* | 186 (28) | 136 (28) | 50 (28) |  | 151 (29) | 35 (27) |  |
| *Other* | 252 (38) | 157 (33) | 95 (53) |  | 199 (38) | 53 (40) |  |
| *(missing, n)* | 2 | 1 | 1 |  | 1 | 1 |  |
| Habitual sleep duration/night |  |  |  | 0·014 |  |  | 0·004 |
| *<7h* | 100 (15) | 59 (13) | 41 (23) |  | 67 (13) | 33 (26) |  |
| *7-7·9h* | 174 (27) | 130 (28) | 44 (25) |  | 142 (27) | 32 (25) |  |
| *8-8·9h* | 266 (41) | 198 (42) | 68 (38) |  | 223 (43) | 43 (33) |  |
| *>9h* | 107 (17) | 81 (17) | 26 (15) |  | 86 (17) | 21 (16) |  |
| *(missing, n)* | 13 | 12 | 1 |  | 10 | 3 |  |
| Chronotype |  |  |  | 0·001 |  |  | <0·001 |
| *Definitely evening* | 6 (0·9) | 4 (0·8) | 2 (1·1) |  | 2 (0·4) | 4 (3·0) |  |
| *Moderately evening* | 78 (12) | 48 (10) | 30 (17) |  | 50 (9·6) | 28 (21) |  |
| *Neither type* | 397 (61) | 281 (59) | 116 (65) |  | 325 (62) | 72 (55) |  |
| *Moderately morning* | 153 (23) | 124 (26) | 29 (16) |  | 129 (25) | 24 (18) |  |
| *Definitely morning* | 20 (3·1) | 19 (4·0) | 1 (0·6) |  | 16 (3·1) | 4 (3·0) |  |
| *(missing, n)* | 6 | 4 | 2 |  | 6 | 0 |  |
| Current health (SF12) |  |  |  | 0·65 |  |  | <0·001 |
| *Excellent* | 57 (9·0) | 44 (9·6) | 13 (7·3) |  | 52 (10) | 5 (3·9) |  |
| *Very good* | 221 (35) | 161 (35) | 60 (34) |  | 199 (39) | 22 (17) |  |
| *Good* | 267 (42) | 187 (41) | 80 (45) |  | 198 (39) | 69 (54) |  |
| *Fair* | 75 (12) | 55 (12) | 20 (11) |  | 51 (10) | 24 (19) |  |
| *Poor* | 15 (2·4) | 9 (2·0) | 6 (3·4) |  | 7 (1·4) | 8 (6·2) |  |
| *(missing, n)* | 25 | 24 | 1 |  | 21 | 4 |  |
| *Data are median (IQR) or n (%) unless otherwise specified. Differences between groups were determined using Pearson’s chi-squared test, Wilcoxon rank sum test, or Fisher’s exact test· ^a^ Multiple shift types could be selected if relevant to the worker’s employment* | | | | | | | |

**Supplementary Table 2**: Comparison of included Raine Study participants against select occupational and education variables from Census data

|  | **Western Australian population Census (2011), ABS data^a^**  (*n*=31,976-31,984)  n (%) | **Employed Raine study participants with sleep data**  (*n*=660)  n (%) |
| --- | --- | --- |
| Income levels ^b^ |  |  |
| *Low* | 19,298 (66) | 322 (51) |
| *Medium* | 7930 (27) | 237 (38) |
| *High* | 1865 (6.4) | 67 (11) |
| *(missing, n)* | 2,891 | 34 |
| Education |  |  |
| *Post-secondary/equal to tertiary* | 13,825 (48) | 316 (48) |
| *(missing, n)* | 3,309 | 24 |
| Occupation ^c,d^ |  |  |
| *Professional/Managerial* | 3,976 (17) | 108 (16) |
| *Clerical* | 3,207 (15) | 112 (17) |
| *Technical/Trade/Labour* | 7,397 (34) | 186 (28) |
| *Other* | 7,324 (34) | 252 (38) |
| *(missing, n)* | 274 | 2 |

Data are presented as number (percentage of available data)

^a^ 2011 census data provided in accordance with previous comparisons between Raine Gen2-22^1^, due to alignment with the time of cohort follow-up. Census denominators vary between ranges shown because of varying numbers of respondents.

^b^ Census categories: Low (≤$35,569), Medium ($35,570 - $61,049), High (≥$61,050). Raine study categories: Low (<$32,000), Medium ($32,000 - $64,999), High ≥($65,000). Category limits vary slightly between datasets due differences in raw income data. Categories calculated after transforming Census data into post-taxation income, based on 2011-2012 tax rates.

^c^ Denominator is employed persons.

^d^ Categories are provided according to the Australian and New Zealand Standard Classification of Occupations, (1^st^ edition, revision one).

**Supplementary Table 3**: Breakdown of shift combinations reported by participants

| **Shift combinations** | **Shift work**  (*n*=180) |
| --- | --- |
| *Days only* | 46 (26) |
| *Nights only* | 15 (8) |
| *Evenings only* | 34 (19) |
| *Days and evenings* | 46 (26) |
| *Days and nights* | 12 (6) |
| *Evenings and nights* | 2 (1) |
| *Days, evenings and nights* | 21 (12) |
| *No shift information* | 4 (2) |

Data are presented as number (percentage of available data)

**Supplementary Table 4**: Adjusted associations between evening and/or night shift work, sleep disorder status and mental health endpoints

|  | **Anxiety** | | | **Depression** | | |
| --- | --- | --- | --- | --- | --- | --- |
| *Predictors* | *β* | *95% CI* | *p* | *β* | *95% CI* | *p* |
| (Intercept) | 2·86 | 2·14 – 3·57 | **<0**·**001** | 3·55 | 2·79 – 4·31 | **<0·001** |
| Shift work (yes) | 0·07 | -0·67– 0·81 | 0·85 | -0·32 | -1·02 – 0·54 | 0·54 |
| Clinical sleep disorder (yes) | 2·39 | 1·63 – 3·16 | **<0·001** | 4·63 | 3·93 – 5·54 | **<0·001** |
| Shift work*Sleep disorder | 2·01 | 0·31 – 3·71 | **0·021** | 0·05 | -1·74 – 1·84 | 0·96 |

*Note: models adjusted for sex, health comorbidities, and typical work hours/week. Anxiety is measured with the GAD-7 (range from 0-21), and depression is measured with the PHQ-9 (range from 0-27). Models reported from n=610 participants (without the 46 participants who reported day work only, and the 4 participants who did not indicate any shift schedules; see Supplementary Table 3).*

**Reference (Supplementary material)**

1. McArdle, N. *et al.* The prevalence of common sleep disorders in young adults: a descriptive population-based study. *Sleep* **43**, (2020).
